# First Case in Lithuania of an Autosomal Recessive Mutation in the *DNAJC30* Gene as a Cause of Leber’s Hereditary Optic Neuropathy

**DOI:** 10.3390/genes16090993

**Published:** 2025-08-23

**Authors:** Liveta Sereikaite, Alvita Vilkeviciute, Brigita Glebauskiene, Rasa Traberg, Arvydas Gelzinis, Raimonda Piskiniene, Reda Zemaitiene, Rasa Ugenskiene, Rasa Liutkeviciene

**Affiliations:** 1 Department of Ophthalmology, Lithuanian University of Health Sciences, Eiveniu Str. 2, LT-50161 Kaunas, Lithuania; liveta.sereikaite@stud.lsmu.lt (L.S.); brigita.glebauskiene@lsmu.lt (B.G.); arvydas.gelzinis@lsmu.lt (A.G.); raimonda.piskiniene@lsmu.lt (R.P.); reda.zemaitiene@lsmu.lt (R.Z.); rasa.liutkeviciene@lsmu.lt (R.L.); 2 Laboratory of Ophthalmology, Neuroscience Institute, Lithuanian University of Health Sciences, Eiveniu Str. 2, LT-50161 Kaunas, Lithuania; 3 Department of Genetics and Molecular Medicine, Lithuanian University of Health Sciences, Eiveniu Str. 2, LT-50161 Kaunas, Lithuania; rasa.traberg@lsmu.lt (R.T.); rasa.ugenskiene@lsmu.lt (R.U.)

**Keywords:** Leber’s hereditary optic neuropathy, c.152A>G, (p.Tyr51Cys) in the *DNAJC30*, mutation, case report

## Abstract

Background: Leber’s hereditary optic neuropathy (LHON) is the most common mitochondrial disorder and an inherited optic neuropathy. Recently, two different LHON inheritance types have been discovered: mitochondrially inherited LHON (mtLHON) and autosomal recessive LHON (arLHON). Our case report is the first diagnosed case of arLHON in a patient of Lithuanian descent and confirms the DnaJ Heat Shock Protein Family (Hsp40) Member C30 (*DNAJC30*) c.152A>G p.(Tyr51Cys) founder variant. Case Presentation: A 34-year-old Lithuanian man complained of headache and sudden, painless loss of central vision in his right eye. On examination, the visual acuity of the right and left eyes was 0.1 and 1.0, respectively. Visual-field examination revealed a central scotoma in the right eye, and visual evoked potentials (VEPs) showed prolonged latency in both eyes. Optical coherence tomography showed thickening of the retinal nerve fiber layer in the upper quadrant of the optic disk in the left eye. Magnetic resonance imaging of the head showed evidence of optic nerve inflammation in the right eye. Blood tests were within normal range and showed no signs of inflammation. Retrobulbar neuritis of the right eye was suspected, and the patient was treated with steroids, which did not improve visual acuity. He later developed visual loss in the left eye as well. A genetic origin of the optic neuropathy was suspected, and a complete mitochondrial DNA analysis was performed, but it did not reveal any pathologic mutations. Over time, the visual acuity of both eyes slowly deteriorated, and the retinal nerve fiber layer (RNFL) thinning of the optic disks progressed. A multidisciplinary team of specialists concluded that vasculitis or infectious disease was unlikely to be the cause of the vision loss, and a genetic cause for the disease was still suspected, although a first-stage genetic test did not yield the diagnosis. Thirty-three months after disease onset, whole-exome sequencing revealed a pathogenic variant in the *DNAJC30* gene, leading to the diagnosis of arLHON. Treatment with Idebenone was started 35 months after the onset of the disease, resulting in no significant worsening of the patient’s condition. Conclusion: This case highlights the importance of considering arLHON as a possible diagnosis for patients with optic neuropathy, because the phenotype of arLHON appears to be identical to that of mtLHON and cannot be distinguished by clinicians.

## 1. Background

Leber’s hereditary optic neuropathy (LHON) is the most common mitochondrial disorder and inherited optic neuropathy [1,2]. Until recently, LHON was thought to be caused exclusively by point mutations in mitochondrial DNA (mtDNA). The three most common mtDNA point mutations are m.3460G>A in MT - ND1 (5–10%), m.11778G>A in MT–ND4 (50–70%), and m.14484T>C in MT–ND6 (15–30%) [3]. The recent discovery of biallelic mutations in a nuclear-encoded gene, DnaJ Heat Shock Protein Family (Hsp40) Member C30 (*DNAJC30*), has shown that there are two distinct forms of LHON inheritance: mitochondrial LHON (mtLHON) and autosomal recessive LHON (arLHON) [4,5,6]. *DNAJC30*-linked arLHON occurs predominantly in the Eastern European population, with the *DNAJC30* gene variant c.152A>G p.(Tyr51Cys) founder variant being by far the most common, accounting for 90% of all disease alleles [4,7]. The other pathogenic variants discovered are c.232C>T; p.(Pro78Ser), c.302T>A; p.(Leu101Gln), and c.230_232del; p._His77del) [4,7]. Kieninger and co-authors also found a variant c.610G>T; p.(Glu204*) in trans with the common c.152A>G; p.(Tyr51Cys) in two affected brothers of Turkish origin [4]. Stenton et al. [5] reported that the prevalence of the homozygous *DNAJC30* p.(Tyr51Cys) variant causing arLHON varies according to the distance from the geographic region of the founder event. In recent studies, the relative proportion of arLHON in all genetically identified LHON patients is 21.0–27.0% in Russia and 1.0–7.7% in Central and Southern Europe [4,5,7]. ArLHON resembles mtLHON and other complex I deficiencies, with incomplete, sex-dependent penetrance predominantly affecting male patients. The male-to-female ratio of 10:1–6:1 in arLHON and 5:1 in mtLHON [4,7,8]. The disease manifests as subacute, simultaneous or sequential, bilateral, painless loss of central vision due to selective degeneration of retinal ganglion cells (RGCs) and their axons [7,9]. All arLHON patients exhibit typical fundus changes in the acute phase: (1) circumpapillary telangiectatic microangiopathy, (2) swelling of the nerve fiber layer around the optic disk (“pseudoedema”), and (3) lack of staining on fluorescein angiography, followed by thinning of the RNFL of the optic disk in the chronic phase, with no extraocular abnormalities observed [4,7,10].

In our manuscript, we present the first diagnosed case of arLHON in a patient of Lithuanian origin, in whom the homozygous *DNAJC30* c.152A>G; p.(Tyr51Cys) founder variant was confirmed.

## 2. Case Report

A 34-year-old man of Lithuanian origin presented to the emergency department with sudden, painless central vision loss in his right eye. At the time of admission, the visual acuity (Snellen chart, Landolt C optotype) of the right and left eyes was 0.1 and 1.0, respectively. Simultaneously, there was a headache on the left side of the head.

**Figure 1:**
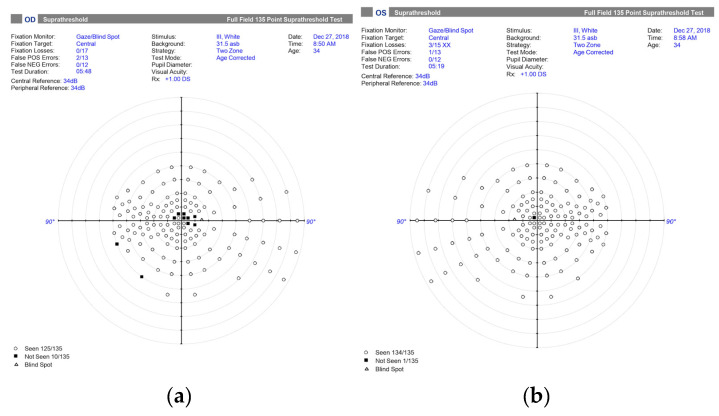
Visual-field testing performed at the onset of sudden, painless central vision loss reveals central scotomas, more pronounced in the right eye. (**a**) Right eye: 10 out of 135 test points not seen, corresponding to a dense central defect; (**b**) Left eye: 1 out of 135 test points not seen, indicating a mild central defect. These findings are consistent with early papillomacular bundle involvement.

**Figure 2:**
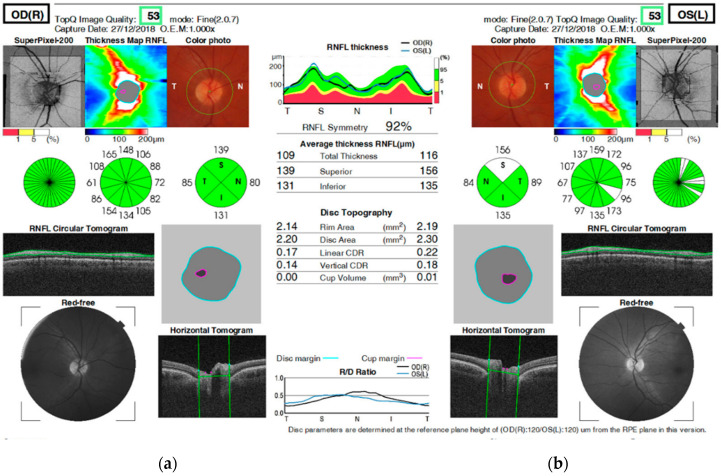
OCT shows RNFL thickening in the superior quadrant of the left eye compared to age-matched normative values (~90–110 µm per quadrant), suggestive of optic disk swelling. (**a**) Right eye: nasal—80 µm, inferior—131 µm, temporal—85 µm, superior—139 µm; (**b**) Left eye: nasal—84 µm, inferior—135 µm, temporal—89 µm, superior—156 µm.

The patient underwent a comprehensive examination. Visual-field testing revealed a central scotoma in the right eye (Figure 1), while visual evoked potentials (VEPs) showed prolonged latency in both eyes. Optical coherence tomography (OCT) revealed thickening of the retinal nerve fiber layer (RNFL) in the superior quadrant of the optic disk in the left eye (Figure 2). Magnetic resonance imaging of the head revealed signs of optic nerve inflammation in the right eye. Blood tests were within normal range and showed no signs of inflammation.

Retrobulbar neuritis of the right eye was suspected, and intravenous methylprednisolone pulse therapy was administered for three consecutive days, followed by oral prednisolone. Treatment did not improve visual acuity in the right eye.

**Figure 3:**
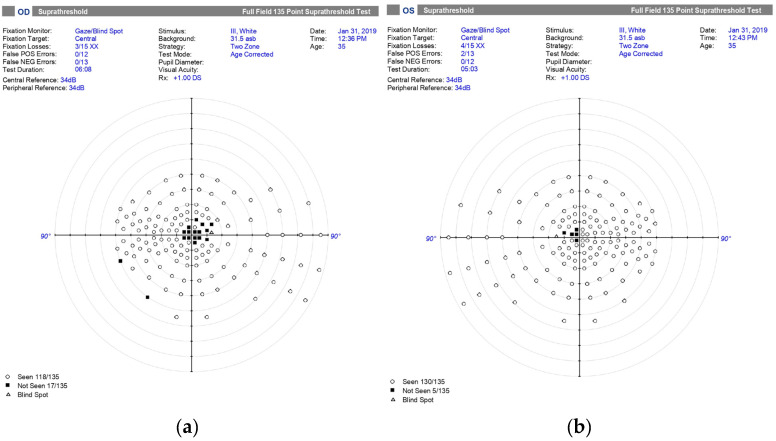
Visual-field testing demonstrates central scotomas in both eyes, more pronounced in the right eye. (**a**) Right eye: 17 out of 135 test points not seen, corresponding to a dense central defect; (**b**) Left eye: 5 out of 135 test points not seen, indicating a milder central defect. These findings reflect progression of papillomacular bundle involvement.

At 5 weeks after onset, the patient developed corresponding painless central visual loss in the left eye. The visual acuity of the left eye decreased to 0.1, with central scotoma in both eyes (Figure 3).

Genetic origin of the optic neuropathy was suspected 6 months after onset. Firstly, the total mitochondrial DNA analysis was performed using next-generation sequencing (NGS) (Method used: polymerase chain reaction and NGS with Illumina MiSeq, computer analysis: BWA2.1 Illumina BaseSpace, Alamut, mtDNA Variant analyser 1000. Tested genes: *MT-ND1*, *MT-ND2*, *MT-ND3*, *MT-ND4*, *MT-ND4L*, *MT-NDS*, *MT-ND6*, *MT-CYB*, *MT-CO1*, *MT-CO2*, *MT-CO3*, *MT-ATP6*, *MT-ATP8*, *MT-RNRI*, *MT-RNR2*, *MT-TA*, *MT-TT*, *MT-TP*, *MT-TE*, *MT-TL2*, *MT-TS2*, *MT-TH*, *MT-TR*, *MT-TG*, *MT-TK*, *MT-TD*, *MT-TS1*, *MT-TC*, *MT-TY*, *MT-TN*, *MT-TW*, *MT-TM*, *MT-TI*, *MT-TQ*, *MT-TL1*, *MT-TF*, *MT-TV*. The analysis did not reveal any pathologenic mutations in the mitochondrial genome. Later, magnetic resonance imaging (MRI) of the brain was repeated, and neurovascular conflict was detected, although the patients’ visual status did not correlate with the described changes.

**Figure 4:**
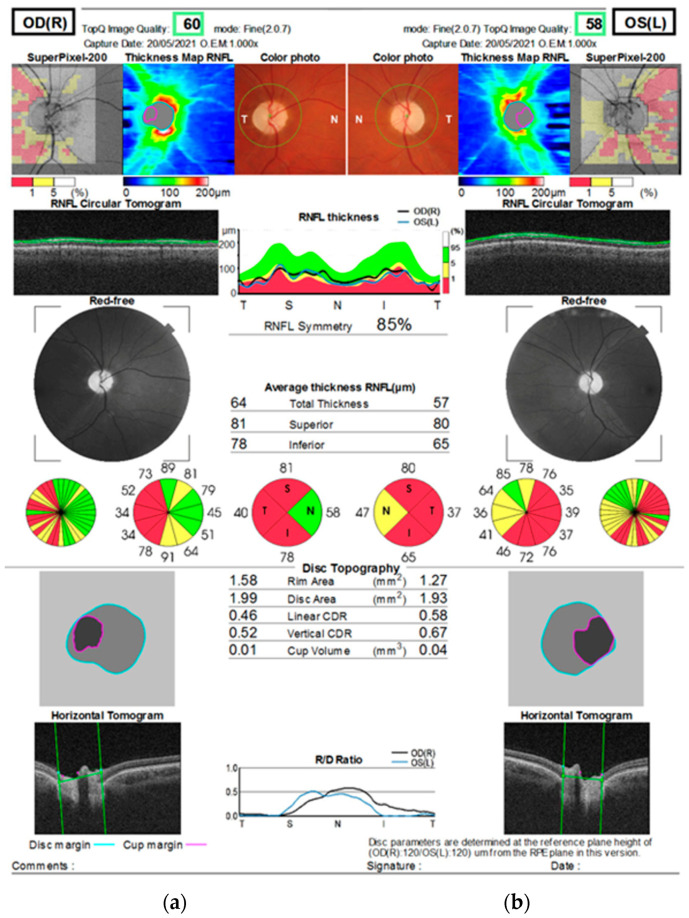
OCT demonstrates marked RNFL thinning in both eyes compared to age-matched normative values (~90–110 µm per quadrant), consistent with bilateral optic nerve atrophy. (**a**) Right eye: nasal—58 µm, inferior—78 µm, temporal—40 µm, superior—81 µm; (**b**) Left eye: nasal—47 µm, inferior—65 µm, temporal—37 µm, superior—80 µm.

**Figure 5:**
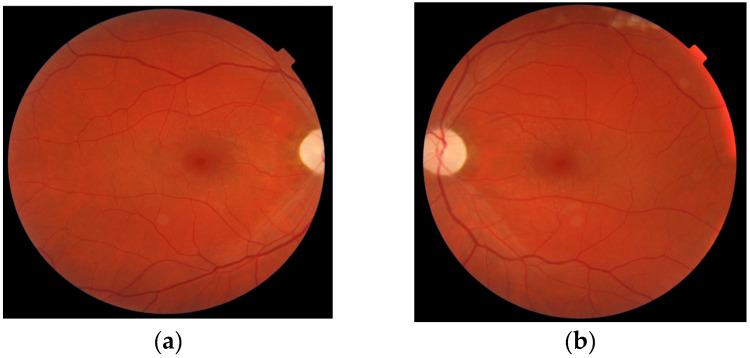
Fundus photography reveals bilateral optic nerve atrophy, with pallor of the optic disks. The central and peripheral retina appear unremarkable, with no evidence of retinal vascular or structural pathology. (**a**) Right eye: optic nerve atrophy; (**b**) Left eye: optic nerve atrophy.

Over time, the visual acuity of both eyes slowly deteriorated, and RNFL thinning of the optic disks progressed. At 30 months after onset, the RNFL thickness of the optic disks in the superior, temporal, and inferior quadrants was below normal limits bilaterally (Figure 4), and the visual acuity of both eyes was 0.06. Fundus examination revealed pallor of the optic disk in both eyes (Figure 5).

A multidisciplinary team of specialists concluded that vasculitis or infectious disease was unlikely to be the cause of the vision loss, and a genetic cause of the disease was still suspected, although first-tier genetic testing did not reveal the diagnosis. Due to limited availability reasons, whole-exome sequencing (WES) using a virtual rare eye disorder gene panel was performed at the age of 37 years (33 months after the onset of the disease). Average sequencing depth—1325. Proprietary software was used for variant detection. Variant calling was performed in the target regions with +/− 30 base pairs. The lists also include variants with low frequencies (OFA down to 2% of sequenced reads). Demultiplexing of the sequencing reads was performed with Illumina bcl2fastq (2.20). Adapters were trimmed with Skewer (version 0.2.2) [11]. Trimmed raw reads were aligned to hg19-cegat using the Burrows–Wheeler Aligner (BWA-mem version 0.7.17-cegat) [12]. The homozygous missense variant c.152A>G; p.(Tyr51Cys) in the *DNAJC30* (NM_032317.3) gene was identified. The variant is classified as pathogenic according to American College of Medical Genetics and Genomics (ACMG) guidelines [13]. The c.152A>G p.(Tyr51Cys) variant frequency in public databases is extremely low. A different amino acid change in the same position is a known pathogenic variant. For a c.152A>G; p.(Tyr51Cys) variant, computational prediction tools unanimously support a deleterious effect on the gene. Pathogenicity of the variant is also confirmed by a functional study [7]. Based on the clinical presentation of the disease, ophthalmologic examination results, and NGS results, a diagnosis of arLHON (ORPHA: 104, OMIM # 619382) caused by the pathogenic variant c.152A>G; p.(Tyr51Cys) in the *DNAJC30* gene was made. Functional confirmation of the pathogenicity of the *DNAJC30* c.152A>G; p.(Tyr51Cys) variant by complex I enzymatic assays or other mitochondrial function testing was not performed in this patient. Given the well-established association of this founder variant with autosomal recessive LHON, and the characteristic clinical presentation, additional functional testing was not deemed essential at the time. However, such testing was considered as part of the initial differential diagnostic work-up.

At the time of diagnosis, the visual acuity of both eyes of the patient was 0.01. Treatment with Idebenone at a dose of 300 mg three times daily was started 35 months after the onset of the disease. In addition to visual acuity, the patient underwent comprehensive longitudinal monitoring of visual function, including visual-field testing (full-field and FDT 10-2), OCT, and electrophysiological assessments VEP and electroretinography (ERG). Visual fields consistently demonstrated central scotomas with preserved peripheral vision and stable mean deviation values, indicating persistent but non-progressive functional deficits over time. OCT imaging revealed progressive thinning of the RNFL and ganglion cell layer, which stabilized after idebenone initiation, supporting structural response to therapy.

Color vision and contrast sensitivity were not assessed using standard tests such as the Ishihara or Farnsworth–Munsell 100 Hue Test due to the patient’s severely impaired vision and inability to perceive colors; thus, these modalities were not tracked over time. VEP recordings showed prolonged P100 latencies, consistent with optic nerve dysfunction, while ERG responses remained within normal limits, confirming preserved outer retinal function.

Overall, the multimodal follow-up highlights the value of structural and functional metrics in monitoring treatment response in arLHON, even when visual acuity alone shows minimal improvement.

Three months after the start of treatment (38 months after the onset of the disease), the results of the examination at OCT were without significant deterioration.

Eleven months after starting treatment (46 months after disease onset), the results of the OCT examination were without significant worsening.

The patient has been receiving idebenone treatment for approximately four years, with intermittent breaks, and continues to undergo regular ophthalmologic monitoring. In line with LHON Consortium guidelines—which recommend continuation of idebenone therapy when partial visual recovery (≥0.1) is observed—a multidisciplinary team comprising ophthalmologists and rare disease specialists decided in April 2024 to extend treatment at 900 mg/day for an additional year. The patient remains under active follow-up, with visual and structural outcomes closely monitored. As of 24 March 2025, visual acuity was stable: monocular visual acuity measured 0.1 in the right eye and 0.09–0.1 in the left eye, with binocular peripheral visual acuity of 0.1. Optical coherence tomography (OCT) demonstrated macular thickness within normal limits (OD 229 µm, OS 236 µm) and no exudation, but showed diffuse thinning of the ganglion cell layer, consistent with optic neuropathy. Visual-field testing (full-field and FDT 10-2) confirmed persistent defects without further deterioration. A timeline summarizing the patient’s clinical course, investigations, and interventions is provided in Table 1.

## 3. Discussion

We present the first diagnosed case of arLHON in a patient of Lithuanian origin caused by the pathogenic founder variant *DNAJC30* c.152A>G; p.(Tyr51Cys).

At disease onset, the patient experienced sudden, painless vision loss, first in one eye and then in the other, which initially raised suspicion for optic neuritis—particularly given the absence of family history and the patient’s age. Early OCT and MRI findings did not show RNFL atrophy or acute inflammatory lesions, although an atypical optic neuritis could not be excluded. Over the following months, progressive bilateral optic nerve atrophy became apparent. Differential diagnoses included dominant optic atrophy, infectious optic neuropathy, vasculitic or autoimmune optic neuropathy, and compressive lesions such as neurovascular conflict. Multidisciplinary evaluations by neuro-ophthalmology, genetics, infectious diseases, neurosurgery, and rheumatology excluded infectious, inflammatory, vascular, and compressive causes, and MRI changes were judged incidental. The diagnostic workup ultimately supported a genetic optic neuropathy, later confirmed as arLHON after genetic re-analysis.

Although arLHON, particularly cases caused by *DNAJC30* mutations, typically presents in late adolescence or early adulthood, our patient developed symptoms at the age of 34—significantly older than the mean age reported in most cohorts. In a large Central European cohort of patients with *DNAJC30*-associated arLHON, the mean age of onset was 18.5 years (range 9.5–45.1 years) [4]. This highlights the clinical variability of arLHON and supports the recognition of late-onset cases. The initial unilateral onset with headache further deviated from the classic LHON phenotype. Our case also supports the growing recognition that arLHON may have a broader clinical spectrum, with atypical age of onset and variable initial symptoms that can mimic other optic neuropathies. Clinicians should therefore maintain a high index of suspicion and consider comprehensive genetic testing even in patients outside conventional diagnostic criteria, especially in regions where such cases have not been reported.

WES was not performed until 33 months after symptom onset due to the limited availability of clinical genetic testing at the time. In March 2019, mitochondrial DNA (mtDNA) genome testing was performed because LHON was suspected, but no pathogenic mutations were identified. In 2020, the Genetic Laboratory of Rare Diseases recommended next-generation sequencing of genes associated with optic nerve atrophy. When the patient’s data were reanalyzed in 2021, the causative *DNAJC30* mutation had been described in the literature, enabling a definitive diagnosis and initiation of targeted treatment. The delay in diagnosis was therefore attributable to evolving genetic testing capabilities and the fact that the mutation had not yet been reported when the patient was first investigated.

As for the pathogenesis of LHON, it is known that complex I (NADH–ubiquinone oxidoreductase) is crucial for respiration in many aerobic organisms and, with 45 subunits, is the first and largest of the electron transfer complexes in the mitochondrial respiratory chain [14,15,16,17]. Complex I is encoded by both mtDNA and nuclear DNA. In mtLHON, mitochondrial DNA mutations affect the subunits responsible for complex I assembly, thus affecting not only complex I assembly but also its stability and activity [18,19,20,21,22]. In contrast, in arLHON, *DNAJC30* mutations play a role in the turnover process of the subunits of the N-module of complex I during the complex I repair mechanism. Recent studies suggest that the damaged subunits of the NADH-oxidizing N-module of complex I, which is subjected to higher oxidative damage and is the primary site of complex I reactive oxygen species production, are rescued and replaced independently of the rest of the complex and at a much higher rate by the mitochondrial matrix protease ClpXP. This requires much less energy than de novo synthesis and reconstruction of the entire complex I [23,24]. Meanwhile, Stenton et al. [7] classified the subunits of complex I into three categories according to their respective turnover rates: CIHIGH (NDUFV3, NDUFS4, NDUFS6, NDUFA6, and NDUFA7), CIMOD, which together constitute the N-module of complex I, and CILOW, which represents the remainder of complex I. They proposed that DNAJC30 is a chaperone protein that degrades the subunits of the N-module of complex I at a high turnover rate and allows CLPXP to access the degradation of subunits with moderate turnover. This facilitates the resynthesis and replacement of subunits of the N-module without the need for complete degradation and synthesis of complex I with high energy expenditure. They demonstrated that in arLHON, in contrast to mtLHON, pathogenic *DNAJC30* variants do not affect the abundance of complex I, but lead to impairment of the complex I repair mechanism and accumulation of complex I with impaired function [7].

According to the reports of previous studies, the manifestation of the disease in this case resembled mtLHON and other complex I deficiencies, and the clinical phenotype of arLHON appeared to be similar to that of mtLHON and indistinguishable by physicians [4,5,7]. However, other authors noted that in arLHON, the age of onset was earlier and shorter (mean 18.5–19.9 years), bilateral manifestation was more common (40.0–46.7%), and clinically relevant recovery of visual impairment was more common without (45.0–69.0%) and with (77.0–80.6%) idebenone treatment compared with mtLHON [4,5,7]. This was not the case in our patient, in whom the disease started at age 34 years and manifested sequentially in one eye and 5 weeks later in the other. Moreover, a clinically relevant improvement in visual impairment is still not observed in both eyes. The fact that he did not start idebenone treatment until 35 months after the onset of the disease or the short follow-up period (6 months) could be the reasons for the lack of clinically relevant recovery of visual function. However, Kieniger et al. [4] reported that in their study, all but one patient were treated six or more months after disease onset and clinically relevant recovery of visual acuity was observed in 45% of cases (*n* = 9) after a median of 19 months, so the timing of treatment initiation may not be the cause of the poor results in our case. At least in mtLHON, younger age at disease onset is associated with a better prognosis. This may account for the poor outcome in our patient, in whom the first symptoms appeared at the age of 34 years, relatively late [25].

Numerous studies have been conducted on the treatment of LHON to find the best option. Different classes of drugs have been investigated, namely mitochondrial electron transport modulators, apoptosis inhibitors, gene therapy, and drugs that can induce retinal tissue regeneration [26]. Idebenone is the first and only approved drug for the treatment of mtLHON. Its mechanism of action is to stimulate ATP production, enhance antioxidant activity, and consequently reduce free radicals generated by damage to complex I of the mitochondrial respiratory chain [27]. In studies by Stenton and co-authors, it was observed that treatment with idebenone was more effective than treatment with mtLHON in patients with a mutation in the *DNAJC30* gene [5,7]. A significant reduction in the time from nadir to recovery was observed. Of the 30 patients treated with idebenone, 77% had CRR in at least one eye. Of the 16 untreated arLHON patients, spontaneous CRR occurred in 69%. Outcomes were better in both groups than in mtLHON patients. The dosage and timing of treatment were determined individually by the treating physicians. In our clinical case, the patient is treated with 300 mg three times daily, but the initial lack of a genetic diagnosis—not drug availability—accounted for treatment delay, and no other mutation-specific interventions were available at the time.

In conclusion, this case report describes the first diagnosed instance of arLHON in a patient of Lithuanian descent and confirms that the *DNAJC30* c.152A>G; p.(Tyr51Cys) founder variant is by far the most prevalent in Eastern European and other populations. This finding underscores the likelihood that arLHON is underdiagnosed in certain regions and highlights the need to consider autosomal recessive inheritance in patients with LHON-like presentations, particularly in areas where only mtDNA-related LHON has been reported. Furthermore, our case reinforces that the clinical phenotype of arLHON is indistinguishable from that of mtLHON, making genetic testing essential. Accordingly, the *DNAJC30* gene should be included in diagnostic panels for optic neuropathies.

## Figures and Tables

**Table 1:** Timeline of clinical course, investigations, and interventions.

Date	Event
December 2018	Onset of visual symptoms, Retrobulbar neuritis of the right eye was suspected, and intravenous methylprednisolone pulse therapy was administered for three consecutive days, followed by oral prednisolone.
March 2019	DNA extracted; mtDNA genome testing performed (suspected LHON)—no mutations detected
December 2020	NGS panel for optic nerve atrophy-related genes
June 2021	Reanalysis of genetic data; pathogenic autosomal recessive *DNAJC30* mutation identified
August 2021	Diagnosis of arLHON confirmed
From October 2021	Idebenone treatment initiated shortly after genetic confirmation, 300 mg three times a day (900 mg daily)

## Data Availability

All the data supporting our findings are contained within the manuscript.

## Abbreviations

The following abbreviations are used in this manuscript:

ACMG	American College of Medical Genetics and Genomics
arLHON	autosomal recessive LHON
LHON	Leber’s hereditary optic neuropathy
mtLHON	mitochondrial LHON
NGS	next-generation sequencing
OCT	Optical coherence tomography
RGCs	retinal ganglion cells
RNFL	retinal nerve fiber layer
WES	whole exome sequencing
